# Large language models for extraction of OPS-codes from operative reports in meningioma surgery

**DOI:** 10.1007/s00701-025-06631-3

**Published:** 2025-07-31

**Authors:** Sebastian Lehmann, Florian Wilhelmy, Nikolaus von Dercks, Erdem Güresir, Johannes Wach

**Affiliations:** 1 Department of Neurosurgery, University Hospital Leipzig, 04103 Leipzig, Germany; 2 Medical Management, University Hospital Leipzig, 04103 Leipzig, Germany

**Keywords:** OPS-Coding, Artificial intelligence, GPT, Meningioma surgery

## Abstract

**Background:**

In the German medical billing system, surgical departments encode their procedures in OPS-codes. These OPS-codes have major impact on DRG grouping and thus mainly determine each case´s revenue. In our study, we investigate the ability of the Large Language Model (LLM) GPT to derive correct OPS codes from the surgical report.

**Methods:**

For our study, 100 patients who underwent meningioma surgery at our clinic between 2023 and 2024 were examined. We recorded the OPS codes assigned by the surgeon after the procedure, as well as the final coding by the hospital´s coders before case closure. In addition, the surgical report was extracted and anonymously provided to GPT-4o and GPT CodeMedic together with the current OPS-catalogue. The coding of each group was analyzed descriptively and compared using the Chi-Square test. Additionally, errors and deviations were assessed and analyzed.

**Results:**

In our analyses, coders (100%) and surgeons (99%) demonstrated to significantly perform higher than LLMs in *sufficient coding*, for which the basic coding must be correct and unquestionable (GPT-4o 78%, GPT CodeMedic 89%; *p* < 0.01). For *optimal coding*, where every code potentially contributing to increase the revenue must be included, only the coders (94%) achieved superiority (GPT-4o *p* < 0.01; GPT CodeMedic *p* = 0.02), whereas GPT CodeMedic (83%) even outperformed surgeons (69%) (*p* = 0.03). The specialized GPT CodeMedic tends to show fewer hallucinations compared to GPT-4o (7% vs. 15%).

**Conclusion:**

GPT is capable of extracting OPS codes from surgical reports. The most frequent errors made by LLMs can be attributed to a lack of specialized training. Currently, professional coders still significantly outperform LLMs in *sufficient* and *optimal* coding. For *optimal* coding however, GPT shows to perform comparably to surgeon´s coding skills. This indicates, that in near future after further training, LLMs may take over this task from surgeons without loss in quality.

**Graphical abstract:**

Large language models for extraction of OPS-codes from operative reports in meningioma surgery

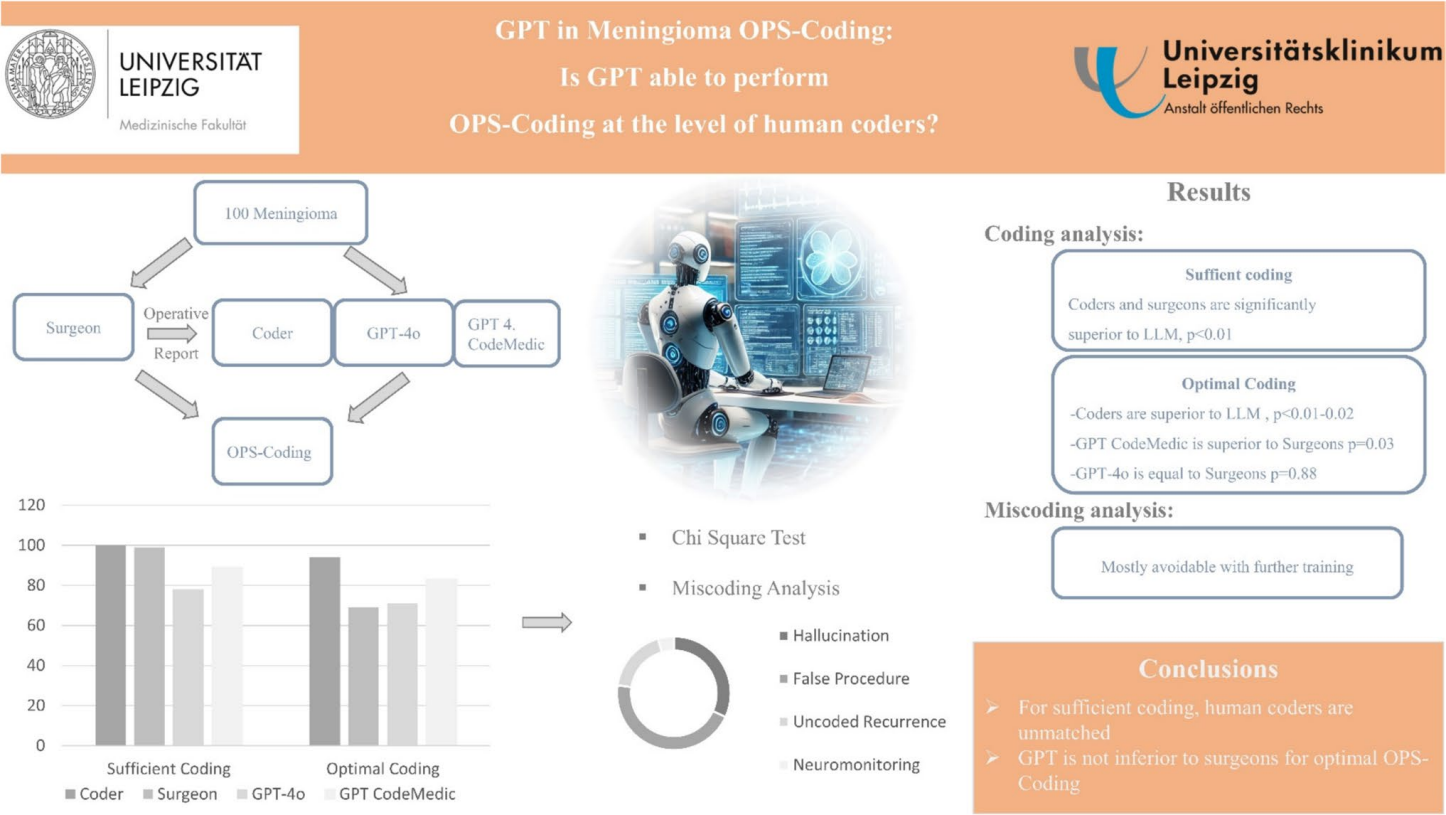

**Supplementary Information:**

The online version contains supplementary material available at 10.1007/s00701-025-06631-3.

## Introduction

The German system for accounting revenue for hospital services is based on classification of cases into “Diagnosis Related Groups” (DRGs). Each DRG is based on a multitude of patient-specific and disease-specific factors [12, 18, 21]. In surgical disciplines, the procedure performed mainly contributes to the classification in the respective DRG. These procedures are coded using OPS-codes that base on the OPS catalogue, annually published by the Federal Institute for Drugs and Medical Devices (BfArM) [7, 13].

In hospital routine, correctly coding a patient’s case is a multi-step procedure. The initial OPS coding is performed by the surgeon that carried out the operation. This initial coding is implemented into the DRG by the hospitals grouping system and cross-checked by the hospital´s coding professionals. Before revenue can be paid, a subset of cases is reviewed by the MDK (Medical Service of Health insurance providers). If incorrect coding detected, it may lead to a reduction in the case value and a fine. Thus correct and efficient coding is a vital process in clinical routine [11].

At a time when medical practice is highly bureaucratized, artificial intelligence is increasingly finding its way into clinical practice [29]. AIs that are able to analyze complex information, understanding context and processing content may be able to make our everyday life in hospitals more efficient, less bureaucratic and more patient-oriented in the future [2, 5, 30].

First studies testing LLMs coding abilities were recently published [25]. While we see an improvement in coding while the LLM progress [16], first studies indicate a superiority of GPT in ICD (international classification of diseases)- coding compared to humans and established coding systems [1, 28].

To our knowledge, this study is the first to analyze the capability of GPT extracting OPS codes from brain tumor related operational reports.

## Methods

### Study design

In this retrospective study, we analyzed 100 surgical reports of patients who underwent meningioma resection (ICD D32.0) in our clinic in the time from January 2023 to December 2024. Sphenoorbital, spinal, intraventricular meningiomas and tumors of the posterior fossa were not included.

### Human and artificial coders of surgical reports

We assessed the four groups for comparison of coding accuracy: 1. The surgeon assigning OPS codes to the performed procedure. 2. The professional coders revising the coding before closing the case. 3. The LLM GPT in official Version GPT-4o [23]. GPT in the custom-refined version “CodeMedic”, that was published in 2023 [8]. This GPT Version has been trained on medical datasets and fine-tuned by supervised learning specialising in medical ICD and OPS coding At the time of our analysis, GPT CodeMedic was uniquely described to be optimised for processing OPS coding.

### Endpoints

For our analysis we defined two endpoints. We defined"sufficient coding", that must contain a correct procedure code (e.g. 5–015.3, removal of tumor of the meninges without infiltration of adjacent tissue), as well as a correct approach code (e.g. 5–010.11, craniotomy via the midline). Additional codes for the description of accessory devices and techniques (e.g. 5–984, microsurgical procedure, 5–988 neuronavigation or 8–925 neuromonitoring) are optional [6]. No additional code may be specified that is not explicitly described in the OR. Thus, sufficient coding includes basic coding that cannot be questioned but potentially does not utilize the revenue of the procedure."Optimal coding"however includes the correct representation of the procedure code, the approach code and all additional codes that comprehensively describe the procedure. Likewise, no codes may be used that are not explicitly described in the surgical report. After extraction of the surgeons and coders coding from each file’s records, the anonymized operational report was provided to GPT (exemplary Chat prompt see suppl. Figure 1). To support GPT´s context understanding, the OPS-Catalogue and our internal list of frequently used codes were provided [6] (suppl. Table 1/2). In the time period from 2022 to 2025, no changes relevant for our research were made in the OPS-catalogue [20]. We generated a prompt instructing GPT to extract a list of the correct OPS-Coding from the operational report. In case where neuromonitoring was used, an additional note was provided as the use and duration is not documented as standard in our operative reports (Suppl. Figure 1). The consultation of GPT took place in February and March 2025.

### Statistics

For analyses, the data were anonymously dichotomized into correct or incorrect extraction and recorded in a computerized database. We carried out further analyses using SPSS (IBM Corp., IBM SPSS Statistics for Windows, Version 29.0.2.0, Armonk, NY: USA) for descriptive analyses of coding capabilities and error distributions. Subsequently, group comparisons were performed for statistical significance using chi-square testing (two-sided* p*-values are reported). Additionally, the hallucination rates of the LLMs were assessed.

## Results

We analyzed a total of 100 surgical reports. Tumor locations were distributed as follows: 44 convexity meningiomas (including 6 in the central region), 30 sphenoid wing meningiomas, 10 falx meningiomas, 9 located at the sphenoid plane or orbital roof, 6 in the olfactory groove and 1 at the tuberculum sellae. Two operations were performed for tumor recurrence.

**Fig. 1:**
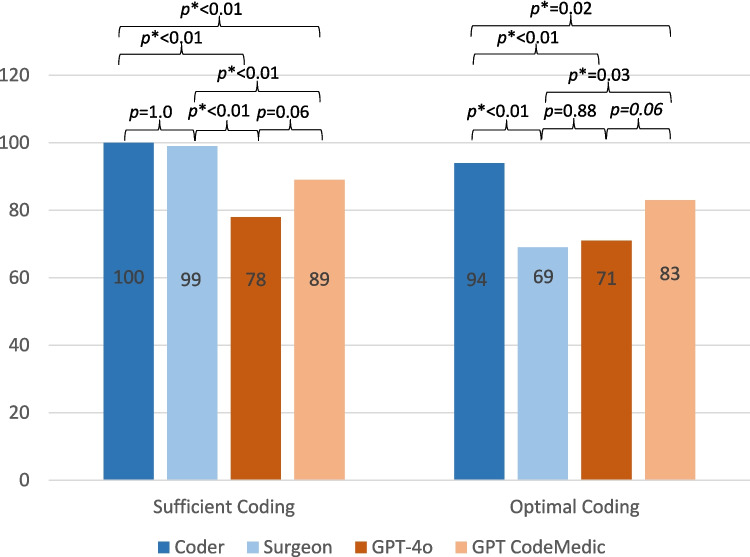
Illustration of sufficient and optimal coding for patients operated on with ICD32.0 (benign meningeal tumor); Sufficient coding: Coder vs. GPT-4o *p* < 0.01; Surgeon vs. GPT-4o *p* < 0.01; Coder vs. GPT CodeMedic *p* < 0.01; Surgeon vs. GPT CodeMedic *p* < 0.01; Coder vs. Surgeon p = 1.0; GPT-4o vs. GPT CodeMedic *p* = 0.06. Optimal coding: Coder vs. GPT-4o *p* < 0.01; Surgeon vs. GPT-4o *p* = 0.88; Coder vs. GPT CodeMedic *p* = 0.02; GPT CodeMedic vs. Surgeon *p* = 0.03; Coder vs. Surgeon *p* < 0.01; GPT-4o vs. GPT CodeMedic *p* = 0.06

*Sufficient* coding was achieved in 99–100% by surgeons and professional coders compared to 78–89% by the LLMs. GPT CodeMedic outperformed GPT-4o by more than 11% in this category. For *optimal* coding, professional coders achieve the highest performance (94%), while surgeons showing the highest error rate (69%). Again, GPT CodeMedic outperformed GPT-4o by more than 10% (Fig. 1).

In cross-tabulation analysis, professional coders were significantly superior to both LLMs in *sufficient* (*p* < 0.01) and *optimal* coding (GPT-4o: *p* < 0.01; GPT CodeMedic: *p* = 0.02). Surgeons showed significant superiority in *sufficient* coding (*p* < 0.01), but performed significantly worse in *optimal* coding. In this category, GPT CodeMedic was even significantly superior to surgeons (GPT-4o: *p* = 0.88; GPT CodeMedic: *p* = 0.03), as well as Coders (*p* < 0.01). In sufficient coding, the performance of surgeons did not differ significantly from GPT-4o (*p* = 0.88). For *optimal* as well as *sufficient* coding, GPT CodeMedic performs better than GPT-4o, with statistical significance only narrowly missed (*p* = 0.06) (Fig. 1; Suppl. Table 3).

**Fig. 2:**
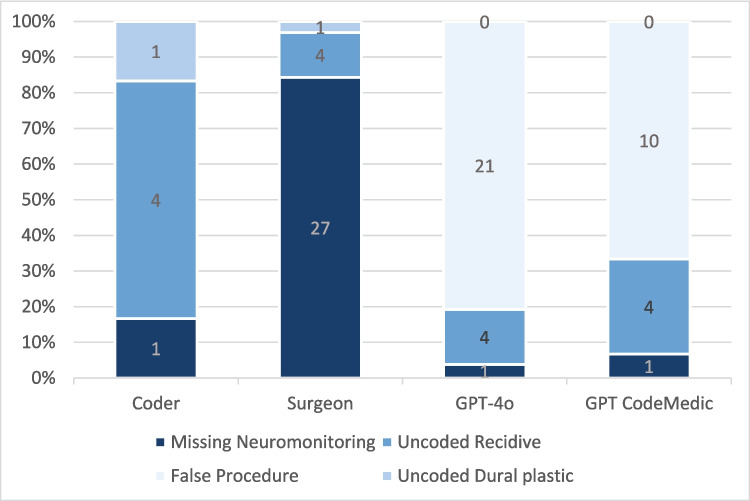
Illustration of the error distribution in the coding for patients operated on with ICD D32.0 (benign neoplasm of the meninges)

Error source analysis revealed distinct patterns between human and machine coders. For surgeons, the most common error was the inaccurate or missing coding of the neuromonitoring (84%). For LLMs, most errors resulted from miscoding of procedures (66–80%). Among professional coders, the most frequent errors were uncoded tumor recurrences. However, this and missing coding of duroplasties (3%) hat minor overall impact. Hallucinations were observed in 7% (GPT CodeMedic) to 15% (GPT-4o), contributing notably to the incorrectly coded procedures (Fig. 2).

In the present analysis including a dataset of 100 operative cranial meningioma procedures, each requiring approximately three minutes of a senior physician’s time for accurate coding. This totals 300 min, or five hours of labor. With a monthly salary of €9,302.27 (TVÄ3-Stage 1) and an estimated 160 working hours per month, the hourly wage of a senior physician is approximately €58.14. Consequently, the total cost incurred for manual coding across 100 procedures amounts to €290.70 in physician time (Table 1). In a high-volume university hospital, where approximately 300 brain tumor surgeries are performed each year, this results in a total of 900 min or 15 h of specialized labor annually. Therefore, the direct labor cost of manual coding for 300 patients amounts to €872.10 per year in a high-volume neurosurgical center. Calculated for procedures in a mid-sized department with approximately 1800 surgical Interventions/year the costs add up to €5,232.6.

**Table 1:** Estimated cost savings from automated coding for the present meningioma cohort

Item	Value
Number of procedures	100
Time per procedure (manual coding)	3 min
Total time saved	300 min (5 h)
Senior physician monthly salary (TVÄ3-Stage 1)	€9,302.27
Estimated monthly working hours	160 h
Hourly wage	€58.14
Total labor cost saved	€290.70

Table 1: Potential cost savings from automated coding for the present meningioma cohort and in a high-volume brain tumor center

## Discussion

Our analysis shows a clear superiority of the professional coders to GPT in both *sufficient* and *optimal* coding. Surgeons however exhibit a 30% gap between *sufficient* and *optimal* coding. Here the surgeons do not perform better than GPT-4o, on the contrary, GPT CodeMedic even achieves statistical superiority.

The error analysis helps to explain these findings. While coders achieve near-perfect results, the errors of surgeon´s and LLM´s are attributable to divergent causes. Coding for meningioma resection is generally straightforward and standardized. Common sources of error—such as recurrence coding—are less frequent in this context.

The most common error for surgeons coding meningioma resections was the correct coding of the neuromonitoring used. However, as this is specifically documented in the casefile, these errors were almost always recognized and corrected by the professional coders.

For LLMs, the most common error was the incorrect application of a code for cranioplasty. This appears to be triggered by a detailed description of bone-flap reinsertion. Tough not entirely being hallucinatory, as the code itself exists, it is wrongly addressed rendering the coding invalid as it is challengeable by the MDK. This illustrated a key limitation of the LLM, where they are not able to distinguish subtle but coding relevant distinctions in surgical language. Here, precise descriptions in the operational reports are vital to prevent miscoding.

Another issue observed is that of true hallucination, where fictional yet superficially plausible codes like: “5–830.4: Haemostasis with bipolar coagulation” are invented by the model. This phenomenon is especially prominent when a LLM assesses a task it is not specifically trained for [3, 4], The possible reduction of hallucination by training may be depicted by our data, as GPT CodeMedic, that was modified to improve interaction with medical coding, especially the German OPS-coding system, shows fewer hallucinations (7%) than the untrained GPT-4o (15%).

LLM use in medical coding is a rapidly evolving field which was still in its early stages only a few years ago [9]. 2024 Soroush et al. [27] published a large series of several LLM performing code extraction for medical diagnosis across different medical coding systems showing results from 1.2–45.9% exact match rates, identifying GPT as the best performing LLM. The ICD code extraction abilities were shown to be improved by provision of additional context enhancement in terms of lead term definition or RAG-based code assignment [15, 24]. If the code extraction was not performed from a digital patient file but from a single report such as discharge summaries, results with high rates of accuracy from over 90% were achieved [24, 28].

While the extraction of ICD 10-based diagnosis coding has been well documented in studies [27], there are currently only a few studies on the extraction of procedure codes. Lehnen et al. [17] show that GPT was able to extract procedural data from operative reports. In comparison to diagnostic code extraction, procedure code extraction showed to be associated with a significantly higher error rate [22]. With the use of one comprehensive report and context enhancement we combine previously identified factors increasing coding effectiveness. The individual effect of these measures however is unclear. To what extent our findings can be generalized to other countries'coding systems remains unclear, as our study uniquely evaluates OPS-based code extraction in the relatively unexplored field of LLM-based procedure code analysis in Germany.

It is likely, that with advancing technology as well as the use of tailored approaches including context enrichment, decision pathway instructions and self-review feedback loops, GPTs accuracy will rise further. This may contribute to the reduction of the bureaucratic burden on a surgeons shoulders like hinted by Dubinski et al., in whose study LLMs successfully created operative reports and discharge letters [10]. In Germany, there are several automated coding tools that can generate the appropriate coding for billing based on machine-readable documents from the hospital information system. What they have in common is that procedures (OPS) are less easily recognised than diagnoses (ICD). This is probably due to the simpler assignment of diagnosis and code, but also to the problem of the heterogeneous description of procedures in the original document.

Our results emphasize to main keys: Surgeon’s coding accuracy may be greatly improved by creating awareness for frequently overlooked codes. LLMs however perform at a level comparable to or exceeding surgeons in optimal coding. However, sophisticated and precise domain-specific training appears to be the key to further improve GPTs coding abilities before being introduced into clinical practise. Further studies are required to examine the transferability of the results to other fields of operation and to other classification systems, such as the German-language Swiss CHOP system [26], the Austrian BMSGPK-based system [19] or the ICD-10-PCS system used in the United States [14].

By even implementing conventional software that automates this coding process, the annual 15 h of highly qualified personnel time could be reallocated to more complex clinical tasks or patient care, translating directly into financial savings. Although this estimated example covers only 300 min, the cumulative savings become significantly more impactful when scaled to larger patient volumes including other procedures (spinal, functional, peripheral nerve, trauma and neurovascular surgery) or longer time periods. Additionally, automated systems offer advantages in standardization, reduced error rates, and faster throughput. This underscores the value of investing in AI-driven coding tools, especially in high-volume surgical specialties such as neurosurgery, where accurate and efficient documentation is directly tied to DRG-based reimbursement and administrative workload reduction.

## Conclusion

Our study shows that LLM possess substantial potential in extracting procedure depicting codes from operational report. However, sophisticated training is mandatory to eliminate unnecessary mistakes and hallucinations. We believe that with more comprehensive training OPS Coding can be a robust target for LLM to relieve surgeons of bureaucratic tasks in clinical routine.

## Supplementary Information

Below is the link to the electronic supplementary material.

Supplementary file1 (DOCX 23.3 KB)

## Data Availability

The datasets generated during the current study are available from the corresponding author on reasonable request.

## Abbreviations

GPT: Generative Pre-trained Transformer
LLM: Large language model
OPS: Operationen und Prozedurenschlüssel
ICD: International Statistical Classification of Diseases
