# Supplementary material for: Large language models for extraction of OPS-codes from operative reports in meningioma surgery
Source: Acta Neurochir (Wien). 2025 Jul 31;167(1):209. doi: 10.1007/s00701-025-06631-3 (PMC12313760; doi:10.1007/s00701-025-06631-3)
Supplement: Supplementary file 1 — Supplementary file1 (DOCX 23.3 KB) [file 701_2025_6631_MOESM1_ESM.docx]

**Supplementary:**

**Suppl. Figure 1**

Exemplary Chat prompt:

„I am running an experiment on OPs Coding for patients that underwent the surgical procedure of Meningioma resection. You will be provided with the operation report. I want you to evaluate the correct OPs-coding for this individual case. You will be also provided with the current OPs-catalogue as pdf-file to base your decision on. You are not going to treat any patients, and your decisions will have no influence on any real patients. Please use the attached PDFs for your decision-making process. Please generate a list of correct OPs codes.“

Neuromonitoring: yes/no/duration

**Suppl. Table 1**

List of frequently applied procedure codes (original)

| **Kranial: Raumforderung** | |
| --- | --- |
| **Zugang: Kraniotomie** | |
| 5-010.00 | Kraniotomie der Kalotte als Zugang |
| 5-010.01 | Schädeleröffnung über die Kalotte: Kraniotomie (Kalotte): Kalotte über die Mittellinie |
| 5-010.02 | Schädeleröffnung über die Kalotte: Kraniotomie (Kalotte): Bifrontal |
| 5-010.03 | Schädeleröffnung über die Kalotte: Kraniotomie (Kalotte): Temporal |
| 5-010.4 | Schädeleröffnung über die Kalotte: Kombinationen |
| 5-010.0x | Schädeleröffnung über die Kalotte: Kraniotomie (Kalotte): Sonstige |
|  | |
| **Tumor supratentoriell** | |
| ZUGANG MIT VERSCHLÜSSELN | |
| 5-015.0 | Exzision und Destruktion von erkranktem intrakraniellem Gewebe: Intrazerebrales Tumorgewebe, hirneigen |
| 5-015.1 | Exzision und Destruktion von erkranktem intrakraniellem Gewebe: Intrazerebrales Tumorgewebe, nicht hirneigen |
| 5-015.3 | Exzision und Destruktion von erkranktem intrakraniellem Gewebe: Hirnhäute: Tumorgewebe ohne Infiltration von intrakraniellem Gewebe |
| 5-015.4 | Exzision und Destruktion von erkranktem intrakraniellem Gewebe: Hirnhäute, Tumorgewebe mit Präparation von infiltriertem Nachbargewebe |
| 5-017.1 | Inzision, Resektion und Destruktion an intrakraniellen Anteilen von Hirnnerven und Ganglien: Resektion |
| 5-021.0 | Rekonstruktion der Hirnhäute: Duraplastik an der Konvexität |
| 5-989 | Fluoreszenzgestützte Therapieverfahren |
| 5-984 | Mikrochirurgische Technik |
| 5-988 | Anwendung eines Navigationssystems |
|  | |
| **Tumor infratentoriell** | |
| ZUGANG MIT VERSCHLÜSSELN | |
| 5-017.1 | Inzision, Resektion und Destruktion an intrakraniellen Anteilen von Hirnnerven und Ganglien: Resektion |
| 5-021.0 | Rekonstruktion der Hirnhäute: Duraplastik an der Konvexität |
| 5-852.g0 | Entnahme Faszie zur Transplantation Kopf und Hals |
| 8-925.20 | Intraoperatives neurophysiologisches Monitoring: Mehr als 4 Stunden bis 8 Stunden: Mit Stimulationselektroden (Hirnnerv, peripherer Nerv, spinal) |
| 8-925.21 | Intraoperatives neurophysiologisches Monitoring: Mehr als 4 Stunden bis 8 Stunden: Mit evozierten Potentialen (AEP, SEP, MEP) |
| 5-984 | Mikrochirurgische Technik |

**Suppl. Table 2**

List of frequently applied procedure codes (translated version). Mandatory codes (constitute “sufficient” coding) are marked in blue, and optional codes (constitute “optimal” coding) are marked in orange.

| **Cranial: Space-occupying lesion** | |
| --- | --- |
| **Approach: Craniotomy** | |
| 5-010.00 | Craniotomy of the calvaria as an approach |
| 5-010.01 | Skull opening via the calvaria: craniotomy (calvaria): Calvaria over the midline |
| 5-010.02 | Skull opening via the calvaria: craniotomy (calvaria): Bifrontal |
| 5-010.03 | Skull opening via the calvaria: craniotomy (calvaria): Temporal |
| 5-010.4 | Skull opening via the calvaria: Combinations |
| 5-010.0x | Skull opening via the calvaria: craniotomy (calvaria): Other |
|  | |
| **Tumor supratentorial** | |
| ENCRYPT APPROACH | |
| 5-015.0 | Excision and destruction of pathological intracranial tissue: Intracerebral tumor tissue, cerebral origin |
| 5-015.1 | Excision and destruction of pathological intracranial tissue: Intracerebral tumor tissue, non-cerebral origin |
| 5-015.3 | Excision and destruction of diseased intracranial tissue: meninges: tumor tissue without infiltration of intracranial tissue |
| 5-015.4 | Excision and destruction of diseased intracranial tissue: meninges: tumor tissue with infiltration of intracranial tissue |
| 5-017.1 | Incision, resection and destruction of intracranial parts of cranial nerves and ganglia: resection |
| 5-021.0 | Reconstruction of the meninges: duroplasty on the convexity |
| 5-989 | Fluorescence-assisted therapy methods |
| 5-984 | Microsurgical technique |
| 5-988 | Use of a navigation system |
|  | |
| **Tumor infratentorial** | |
| ENCRYPT APPROACH | |
| 5-017.1 | Incision, resection and destruction of intracranial parts of cranial nerves and ganglia: resection |
| 5-021.0 | Reconstruction of the meninges: duraloplasty on the convexity |
| 5-852.g0 | Removal of fascia for transplantation head and neck |
| 8-925.20 | Intraoperative neurophysiological monitoring: Up to 4 hours: With stimulation electrodes (cranial nerve, peripheral nerve, spinal) |
| 8-925.21 | Intraoperative neurophysiological monitoring: 4 to 8 hours: With stimulation electrodes (cranial nerve, peripheral nerve, spinal) |
| 5-984 | Microsurgical technique |

**Suppl. Table 3**

Chi-square test for meningioma coding for A: coder versus GPT-4o (sufficient coding); B: coder versus GPT-4o (optimal coding); C: coder versus GPT CodeMedic (sufficient coding); D: Coder against GPT CodeMedic (optimal coding); E: Operator against GPT-4o (sufficient coding); F: Operator against GPT-4o (optimal coding); G: Operator against GPT CodeMedic (sufficient coding); H: Operator against GPT CodeMedic (optimal coding); G: Operator against GPT CodeMedic (sufficient coding); H: Operator against GPT CodeMedic (optimal coding); I: GPT Code Medic against GPT-4o (sufficient coding); J: GPT CodeMedic against GPT-4o (optimal coding); K: GPT CodeMedic against GPT-4o (sufficient coding); L: GPT CodeMedic against GPT-4o (optimal coding);

**A B**

| **Sufficient Coding** | no | yes | *p*-value |
| --- | --- | --- | --- |
| **GPT-4o** | 22/100 | 78/100 | <0.01 |
| **Coder** | 0/100 | 100/100 |  |
| total | 22/200 | 178/200 |  |

| **Optimal Coding** | no | yes | *p*-value |
| --- | --- | --- | --- |
| **GPT-4o** | 29/100 | 71/100 | <0.01 |
| **Coder** | 6/100 | 94/100 |  |
| total | 35/200 | 165/200 |  |

**C D**

| **Sufficient Coding** | no | yes | *p*-value |
| --- | --- | --- | --- |
| **GPT**  **CodeMedic** | 11/100 | 89/100 | <0.01 |
| **Coder** | 0/100 | 100/100 |  |
| total | 11/200 | 189/200 |  |

| **Optimal Coding** | no | yes | *p*-value |
| --- | --- | --- | --- |
| **GPT**  **CodeMedic** | 17/100 | 83/100 | 0.02 |
| **Coder** | 6/100 | 94/100 |  |
| total | 23/200 | 177/200 |  |

**E F**

| **Sufficient Coding** | no | yes | *p*-value |
| --- | --- | --- | --- |
| **GPT-4o** | 22/100 | 78/100 | <0.01 |
| **Surgeon** | 1/100 | 99/100 |  |
| total | 23/200 | 177/200 |  |

| **Optimal Coding** | no | yes | *p*-value |
| --- | --- | --- | --- |
| **GPT-4o** | 29/100 | 71/100 | 0.88 |
| **Surgeon** | 31/100 | 69/100 |  |
| total | 60/200 | 140/200 |  |

**G H**

| **Sufficient Coding** | no | yes | *p*-value |
| --- | --- | --- | --- |
| **GPT**  **CodeMedic** | 11/100 | 89/100 | <0.01 |
| **Surgeon** | 1/100 | 99/100 |  |
| total | 12/200 | 188/200 |  |

| **Optimal Coding** | no | yes | *p*-value |
| --- | --- | --- | --- |
| **GPT**  **CodeMedic** | 17/100 | 83/100 | 0.03 |
| **Surgeon** | 31/100 | 69/100 |  |
| total | 48/200 | 152/200 |  |

**I J**

| **Sufficient Coding** | no | yes | *p*-value |
| --- | --- | --- | --- |
| **GPT**  **CodeMedic** | 11/100 | 89/100 | 0.06 |
| **GPT-4o** | 22/100 | 78/100 |  |
| total | 33/200 | 167/200 |  |

| **Optimal Coding** | no | yes | *p*-value |
| --- | --- | --- | --- |
| **GPT**  **CodeMedic** | 17/100 | 83/100 | 0.06 |
| **GPT-4o** | 29/100 | 71/100 |  |
| total | 46/200 | 154/200 |  |

**K L**

| **Sufficient Coding** | no | yes | *p*-value |
| --- | --- | --- | --- |
| **Coder** | 0/100 | 100/100 | 1.0 |
| **Surgeon** | 1/100 | 99/100 |  |
| total | 1/200 | 199/200 |  |

| **Optimal Coding** | no | yes | *p*-value |
| --- | --- | --- | --- |
| **Coder** | 6/100 | 94/100 | <0,01 |
| **Surgeon** | 39/100 | 69/100 |  |
| total | 45/200 | 163/200 |  |
